# Teacher profiles in higher education: the move to online education during the COVID-19 crisis

**DOI:** 10.1007/s10984-023-09458-w

**Published:** 2023-03-16

**Authors:** T. M. Stevens, P. J. den Brok, O. Noroozi, H. J. A. Biemans

**Affiliations:** 1 Eindhoven School of Education (ESoE), Eindhoven University of Technology (TU/E), Eindhoven, The Netherlands; 2 Education and Learning Sciences (ELS) Group, Wageningen University and Research, Wageningen, The Netherlands

**Keywords:** COVID-19, Educational innovation, Higher education, Online education, Teacher profiles

## Abstract

During the COVID-19 pandemic, teachers were forced to move their teaching completely online. While some seized the opportunity to learn and innovate, others experienced difficulties. This study provides insights into the differences between university teachers during the COVID-19 crisis. A survey among university teachers (N = 283) was conducted to investigate their attitudes towards online teaching, beliefs about students’ learning, level of stress experienced, self-efficacy and beliefs about their own professional development. Employing a hierarchical cluster analysis, four distinct teacher profiles were found. Profile 1 was critical but eager; Profile 2 was positive but stressed; Profile 3 was critical and reluctant; Profile 4 was optimistic and easy-going. The profiles differed significantly in their use and perception of support. We suggest that teacher education research should carefully consider sampling procedures or take a person-centred research approach and that universities should develop targeted forms of teacher communication, support and policy.

## Introduction

### Teaching during the COVID-19 crisis

The COVID-19 pandemic caused a sudden move to online education for universities around the world (Arday, 2022; Cahyadi et al., 2022; Gazi & Nelson, 2021; Saha et al., 2022). Although there is some knowledge about the factors that contribute to the effective adoption of online teaching methods in higher education (Yadav et al., 2017), the COVID-19 crisis presented an entirely new set of circumstances (Cutri et al., 2020; Gazi & Nelson, 2021; Hodges et al., 2020; Wild et al., 2021). Before the crisis, online tools were often combined with in-class teaching methods to develop a blend of learning environments (Shardlow et al., 2022; Yeo et al., 2006). Innovations were planned and designed carefully in accordance with potential benefits (Yadav et al., 2017). Risks and failures were avoided, with new online teaching tools and learning environments being driven by the ambition to exploit the opportunities of the new technologies (Shardlow et al., 2022; Yeo et al., 2006). Moreover, only a few teachers were involved in experimenting with new ICT tools (‘innovators’ or ‘early adopters’).

During the COVID-19 pandemic, however, change was unplanned and abrupt. There was no other choice for teachers: online technologies offered the only solution to very urgent and definite problems (Cutri et al., 2020). Teaching staff from all backgrounds and of all ages had to prepare and deliver classes remotely, often without adequate technical, educational and organisational support (Hodges et al., 2020; Scherer et al., 2020). The transition thus was largely in the hands of teachers: the quality of online education relied heavily on their online teaching skills and adaptability.

Although much has been published about the implications of COVID-19 for universities (Arday, 2022; Cutri et al., 2020; Zawacki‐Richter, 2020), few peer-reviewed studies have included empirical evidence about university teachers’ perceptions and behaviours during the crisis (Akour et al., 2020; Almazova et al., 2020; Daumiller et al., 2021; Scherer et al., 2020). These studies involved analysing the relationships between various factors, such as attitudes, self-efficacy, stress, perceptions of support, coping activities and background characteristics including age and gender (Akour et al., 2020; Almazova et al., 2020; Scherer et al., 2020). Although these studies generated important knowledge about the relationships between variables, they did not provide a comprehensive understanding of the differences between teachers. Thus, it is unclear how teachers cope differently with the forced move to online education. This is an important research gap, because the COVID-19 crisis forced all teachers, without exception, to move their teaching online, large differences between teachers can be expected. Moreover, teachers’ psychological responses to dealing with the new circumstances are likely to form coherent patterns within individuals—similar to teachers’ coping styles (Herman et al., 2018), teaching styles (Heimlich & Norland, 2002) and personality profiles (Perera et al., 2018). Hence, the diversity of teachers and the wide range of factors involved in the move to online education present a unique opportunity to explore differences between university teachers.

This study’s aim was to provide a comprehensive understanding of the differences between teachers in the move to online education during the COVID-19 crisis, by taking an inductive, person-oriented research approach. Specifically, the goal was to identify teacher profiles that emerged in the move to online education based on psychological variables, and to identify how these profiles are related to the perception and use of services and background characteristics. Teacher profiles can help universities to develop more-targeted forms of teacher support, communication strategies and educational policies.

### Conceptual framework and research questions

Since the outbreak of COVID-19, academics and practitioners have been debating what concepts and theories are best suited to evaluating the changes in education. Some scholars see the transition as an opportunity to investigate online education at a large scale (Zimmerman, 2020). Others emphasise that, because emergency remote teaching (ERT) during the pandemic is different from high-quality online education that is part of planned innovations, we should resist making comparisons (Hodges et al., 2020). This study’s research design did not utilise one specific theoretical framework; it built on recent empirical studies about teachers in higher education during the COVID-19 pandemic, as well as the wider literature about teachers’ responses to innovations involving educational technology and online teaching methods. We did this to identify key factors that could be indicative for differences between teachers in coping with the transition to online education. In this section, we describe: literature about university teachers’ perceptions and behaviours during the COVID-19 ERT period; the wider field of research about (unidimensional) teacher profiles in the context of educational technology and innovation; and literature about multi-dimensional teacher profiles related to coping. We then synthesise these findings and present this study’s rationale and three research questions.

Although there are many reports about teacher surveys at universities (Gazi & Nelson, 2021; VSNU, 2020), peer-reviewed research studies about university teachers’ perceptions and behaviours during the COVID-19 ERT period are still rare. When (Almazova et al., 2020) conducted a survey among 87 university teachers to analyse the challenges that teachers experienced, the main challenges were: the computer literacy level of teachers, the university electronic environment and support, academic staff readiness and students’ readiness for online education. Daumiller et al. (2021) studied teachers, attitudes towards this shift and associations with underlying motivations, burnout, engagement and student learning. Learning approach goals were associated with perceiving the shift to online teaching as a positive challenge and as useful for personal competence development. Conversely, performance avoidance and work avoidance goals were associated with perceiving the change as threatening, which was in turn associated with burnout levels. When Akour et al. (2020) investigated university teachers’ psychological status, challenges faced in distance teaching and coping activities, they found that most teachers had moderate to high motivation for distance teaching, but that they also experienced high levels of stress. Stress showed a weak negative correlation with teacher age. However, differences between teachers were not investigated in greater detail. Scherer et al. (2020) did look into differences between teachers, but they focused on three indicators of online teaching readiness: technological and pedagogical content knowledge (TPACK) self-efficacy, perceived teaching practice and perceived institutional support. They identified three teacher profiles: high, low and ‘inconsistent’ readiness profiles with mixed outcomes (high and low) on the various indicators. Profile membership was explained by several individual and contextual variables, including gender, prior online teaching experience, innovation potential and cultural orientation (Scherer et al., 2020). They concluded that different subgroups of teachers existed, which required different approaches for support. However, although the study was conducted during the COVID-19 first lockdown and 80.7% of the teachers were forced to move their teaching online, the profiles were the result of a model-based profile analysis on indicators of a single dimension (‘online teaching readiness’). They did not consider some of the key factors that shape how teachers cope with emergency remote education, such as experiences of stress and attitudes towards online teaching (Akour et al., 2020; Almazova et al., 2020; Hodges et al., 2020; MacIntyre et al., 2020).

The wider field of research about teacher profiles in the context of educational technology and innovation generally considers more factors, such as teachers’ beliefs about the effect of technology on students’ learning and beliefs about their own professional development (Garone et al., 2019; Jimoyiannis & Komis, 2007; Tao & Rosa Yeh, 2008a, 2008b). However, these studies have often taken a deductive approach to investigating differences between teachers based on a single variable, which led to rather restrictive, unidimensional profiles. These include teachers with negative, neutral and positive perceptions about ICT in education (Jimoyiannis & Komis, 2007), teachers with sceptical, mild-promising and optimistic beliefs about distance education (Tao & Rosa Yeh, 2008a, 2008b) and teachers with low, medium or high acceptance of technology (Garone et al., 2019). These results do not provide a comprehensive understanding of the heterogeneity of the teacher population in response to radical educational changes.

Research that differentiates teachers on multiple dimensions into qualitatively-distinct profiles tends to distinguish personality profiles or teaching styles based on relatively-persistent personality traits that are largely independent of context. For example, based on the big five personality dimensions, research has identified four distinct teacher personality profiles: ‘rigid’, ‘ordinary’, ‘well-adjusted’ and ‘excitable’ (Perera et al., 2018). Similarly, research shows that teachers’ psychological responses to new, challenging situations are configured into coherent patterns and can be used to identify subgroups of teachers. For example, a study about teacher adjustments identified four profiles with different levels of stress, burnout and coping (Herman et al., 2018). A study about teachers’ coping strategies revealed correlations between avoidant or approach strategies and psychological outcomes, such as well-being, stress and emotions (MacIntyre et al., 2020).

In conclusion, the literature suggests that the move to online education during the COVID-19 crisis involved factors related to teachers’ responses to innovations that involve educational technology in general (e.g. attitudes toward online teaching and beliefs about the effects) and factors relevant to ERT specifically (e.g. experienced levels of stress). Despite theoretical differences, studies generally included similar types of variables in their models: (1) psychological variables, such as beliefs, attitude, self-efficacy and stress; (2) perceptions of support, such as the perceived level of support or satisfaction about services; (3) behavioural variables related to teaching practices or professional development, such as self-reported use of ICT tools in teaching and the participation in trainings; and (4) background characteristics of the person, such as gender and age. Teachers’ psychological responses are likely to form coherent patterns within individuals. Our study aimed to identify teacher profiles in the forced move to online education based on psychological variables, and to identify how these profiles relate to the use and perception of services (trainings, tools, support) and background characteristics. Hence, similar to previous comparable profile studies (Herman et al., 2018; Jimoyiannis & Komis, 2007; Scherer et al., 2020), psychological variables were used to identify teacher profiles, and combined with context-specific variables (the use and perception of context specific services) and background characteristics (gender, age). In order to inductively explore differences between teachers, five basic psychological variables were included: attitudes towards online teaching, beliefs about students’ learning in online education, experienced level of stress, digital and didactical self-efficacy and beliefs about their own professional development. Perceptions of support include the perceived level of support and the satisfaction about teacher training, online teaching tools and education support services. The behavioural factors include the use of online teaching tools, participation in teacher training and the use of education support services. Lastly, gender, age and teacher role (course coordinator or lecturer) were included as background characteristics. The aim of this study was to provide a comprehensive understanding of teacher profiles in the move to online education during the COVID-19 pandemic by addressing the following research questions:

1. What type of university teacher profiles can be distinguished in the move to online education during the COVID-19 crisis situation based on attitudes towards online teaching, beliefs about students’ learning, experienced level of stress, self-efficacy and beliefs about professional development?
2. How do the teacher profiles differ in perceptions about support and the use of online teaching tools, teacher trainings and support services?
3. How do the teacher profiles relate to gender, age and role?

## Method

### Survey sample and procedure

This study focussed on teachers at [removed for double blind review]. The questionnaire used for this study was developed in collaboration with the educational support department and covered a wide range of relevant topics, with a limited number of questions for each topic in order to avoid overburdening teachers. Hence, theoretical knowledge about factors that are likely to affect how teachers cope with emergency remote teaching (see conceptual framework) was combined with context-specific knowledge about relevant aspects according to practitioners and our own team of educational researchers to define the key variables and construct corresponding items. The questionnaire was tested by three teachers, a panel of practitioners (of the educational support department and other departments), educational researchers and one methodologist with expertise on survey research. This led to minor adjustments before its dissemination. All teachers who lectured or coordinated a course were approached to participate in the online survey by email. Participants were informed about the study and the provision to fill in the survey anonymously by leaving some items blank. To validate a minimum active role in the respective teaching period, participants were first asked if they spent at least six hours teaching in the given period. This study’s survey data covers teaching experiences from March until July 2020, which is the period when teachers were first forced to teach almost entirely online and had little time to prepare. In total, 289 teachers (21%) participated in the survey.

Teachers were asked to indicate their age, gender and teacher role. The university differentiates between ‘course coordinators’, who are responsible for the course and are involved in many teaching activities, and ‘lecturers’ who teach only part of a course. An analysis of respondents’ background characteristics indicated a representative sample of the teacher population at [removed for double blind review] in terms of age (*N* = 112, *M* = 45, *SD* = 11.26), gender (*N* = 157, 54% male, 46% female) and teaching role (*N* = 272, 60% lecturers, 40% coordinators). There was no significant relationship between background characteristics, except for age and gender (*F*(2109) = 6.50, *p* < 0.001). Female teachers were younger (*M* = 43) than male teachers (*M* = 48).

### Measures

The survey included questions about teachers’ attitude towards online teaching, beliefs about students’ learning, experiences of stress, digital and didactic self-efficacy, beliefs about their own professional development and perceived level of support. For each construct variable, we used multiple items with a five-point answering scale—mostly Likert scales ranging from ‘strongly disagree’ to ‘strongly agree’. The intent was to use a limited number of items for each factor to establish an adequate level of reliability for exploratory research. Teachers’ attitude towards online teaching was measured using three items: how they liked online teaching, their motivation to teach online and the perceived success of online teaching (e.g. “I like online teaching”). Teachers’ beliefs about students’ learning were measured by five items: students’ learning performance, level of feedback, collaborative learning among students, students’ motivation and students’ engagement of students (e.g. “Please indicate how you think the learning of students is affected by online education on a 5-point scale from worse to better”). Teachers’ stress levels were measured by three items: experienced stress, experienced difficulty working from home, and workload (e.g. “I experienced stress teaching this course online”). Self-efficacy was measured by perceived IT/digital and pedagogical/didactical know-how to be able to teach online (e.g. “I have the IT/digital know-how to be able to teach online”). Beliefs about professional development were measured by three items: increased ability to use online tools, increased ability to apply new online teaching methods, and stimulation to rethink the course design (e.g. “Teaching this course online increased my ability to apply (various/new) teaching methods”). Finally, the perceived level of support was measured by four items: IT infrastructure, information and communication to support online teaching, the teaching support services and support from colleagues and the organisation (e.g. “The IT infrastructure of [removed for double blind review] (software, network, etc.) supported my online teaching”). We measured the internal consistency with Cronbach’s alpha test to interpret the reliability of these construct variables (Table 1). None of the items decreased the Cronbach’s alpha of a variable. Self-efficacy had the lowest reliability of 0.64, which is generally considered acceptable for the number of items (Feldt & Charter, 2003) and reliable for exploratory research (Nunnally & Bernstein, 1994; Stanley et al., 2014).

**Table 1:** Statistics of construct variables

Variable	*N* cases	Items	M	SD	Cronbach alpha
Attitude towards online teaching	287	3	3.53	0.83	0.65
Beliefs about students’ learning	269	5	2.43	0.58	0.76
Experienced level of stress	287	3	3.73	0.93	0.70
Self-efficacy	286	2	3.82	0.77	0.64
Beliefs about professional development	286	3	3.65	0.81	0.67
Perceived level of support	287	4	4.01	0.72	0.74

Teachers were also asked about their use of and satisfaction with education support services (11 items), teacher trainings (11 items) and online teaching tools (39 items). The use of a service (support, training or tool) was measured as a binary item ‘yes’ or ‘no’, and overall use was established into a variable based on the sum total ‘frequency of use’ for each type of service. The evaluation of a support service, teacher training and online teaching tool were measured on a five-point satisfaction scale, and the average satisfaction for a type of service was used as a variable (Table 2).

**Table 2:** Statistics of variables

Variable	Cases	Items	Item options	*M*	*SD*
Use of (participation in) teacher trainings	288	11	Binary yes/no	1.42	1.61
Satisfaction with teacher trainings	189	11	Five-point satisfaction scale	3.66	0.90
Use of support services	288	11	Binary yes/no	4.05	2.54
Satisfaction about support services	259	11	Five-point satisfaction scale	3.47	0.87
Use of online tools	288	39	Binary yes/no	6.96	4.02
Satisfaction about online tool	275	39	Five-point satisfaction scale	3.58	0.76

### Cluster analysis

To establish teacher profiles, a hierarchical cluster analysis was conducted on the five psychological variables: attitude towards online teaching, beliefs about students’ learning, experienced level of stress, self-efficacy and beliefs about professional development. A precondition test was performed to confirm the low collinearity and multicollinearity among these cluster variables with Spearman’s rho and Variance Inflation Factor (VIF) (Galloway & Bretz, 2015; Sarstedt & Mooi, 2019)—see appendix 1 and 2 respectively. We then conducted a hierarchical agglomerative cluster analysis on these variables using Ward’s method as the linkage method and squared Euclidian distance for the interval measure. Ward’s (1963) method combines objects, and groups by minimising the sum of squared Euclidean distances between objects, thus minimising within-cluster variance. The Euclidean distance is the square root of the sum of the squared differences in the variables’ values, and it is the most-commonly used metric for clustering analysis on ordinal variables with equidistant scales (Sarstedt & Mooi, 2019).

The agglomerative clustering method was applied to a range of cluster solutions from 2 to 5 clusters, which was expected to produce the most significant and meaningful results for the number of variables (5) and cases (289) in this study. Subsequently, various statistics were used to evaluate and interpret the cluster solutions 2 to 5. ANOVA was applied on each cluster solution (for 2, 3, 4, and 5 number of clusters), using eta squared values as measures of association. The percentage of variability accounted for by the variable indicates the effect of each variable on the cluster. The sum total *F* of each cluster solution was also measured and compared with the preceding and subsequent cluster solution to calculate the Variance Ratio Criterion (VRC) (Galloway & Bretz, 2015; Sarstedt & Mooi, 2019). The output was analysed for communalities, distinctiveness and meaningful interpretation of the clusters, such as the size of clusters (no outlier profiles of a small size), low variance within clusters, and high variance and variation between clusters (Galloway & Bretz, 2015). A hierarchical cluster analysis is an iterative process in which the interpretations of intermediate findings reveal patterns in the data, so the intermediate results and interpretations are reported in Appendix 3. The results of the cluster analysis were discussed with practitioners (e.g. teachers, education support teams and policy officers) to interpret the cluster profiles. Lastly, we developed profile descriptions and names that highlight the distinctive features of each cluster in relation to the other clusters.

### Analysis of the relationships between clusters and other variables

We performed subsequent statistical analyses to understand how the clusters related to background characteristics (gender, age and teaching role) and the use and perception of support. Pearson’s chi square tests were applied to measure the association between the clusters and the nominal variables gender and teaching role. To determine the significance of the pairwise relations between each cluster and category, we calculated z-scores based on the adjusted residual for each pair. We based our conclusions on the premise that an adjusted residual > 1.96 indicates that the number of cases in that cell is significantly larger than would be expected if the null hypothesis were true, with a significance level of 0.05 (Everitt & Skrondal, 2010).

One-way between-groups analysis of variance (ANOVA) with Tukey multiple comparison post hoc test was applied to measure the relationship between the clusters and the other metric variables of: age; perceived level of support; total use of support services; total use of training; total use of tools; average satisfaction about support services; average satisfaction about training; and average satisfaction about tools. Because of the unequal variances and unequal group sizes, we also applied the Levene, Welch and Brown-Forsythe statistic. All variables that showed significance with ANOVA and the Levene test also showed significance with the Welch and Brown-Forsythe tests.

## Results

### Four teacher profiles

The cluster solution of four clusters proved to be the most distinctive and meaningful, with a relatively-equal distribution of cluster sizes, low variance within clusters and high variance and variation between clusters (see Appendix 3 for the full results and interpretations). As can be seen in Table 3, each variable had a significant effect on the cluster solution (*p* < 0.001) with relatively high effect sizes, ranging from 0.3 to 0.57. The number of teachers per cluster was evenly distributed, ranging from 54 to 110 teachers (Table 4).

**Table 3:** ANOVA for four-clusters solution

Variable	Variance	Sum of squares	*df*	Mean square	*F*	*p*	η	η2
Attitude towards online teaching	Between groups	111.16	3	37.05	124.98	0.00	0.76	0.57
	Within groups	82.71	279	0.30				
	Total	193.87	282					
Beliefs about students’ learning	Between groups	39.59	3	13.20	66.63	0.00	0.65	0.42
	Within groups	55.26	279	0.20				
	Total	94.85	282					
Experienced level of stress	Between groups	99.57	3	33.19	64.43	0.00	0.64	0.41
	Within groups	143.73	279	0.52				
	Total	243.30	282					
Beliefs about professional development	Between groups	66.63	3	22.21	51.65	0.00	0.60	0.36
	Within groups	119.98	279	0.43				
	Total	186.61	282					
Self-efficacy	Between Groups	50.48	3	16.83	39.72	0.00	0.55	0.30
	Within Groups	118.19	279	0.42				
	Total	168.67	282

**Table 4:**
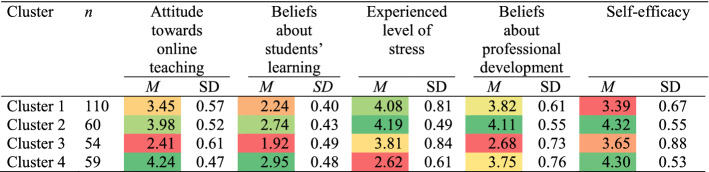
Number of teachers and mean and standard deviation for five variables for each cluster

**Fig. 1:**
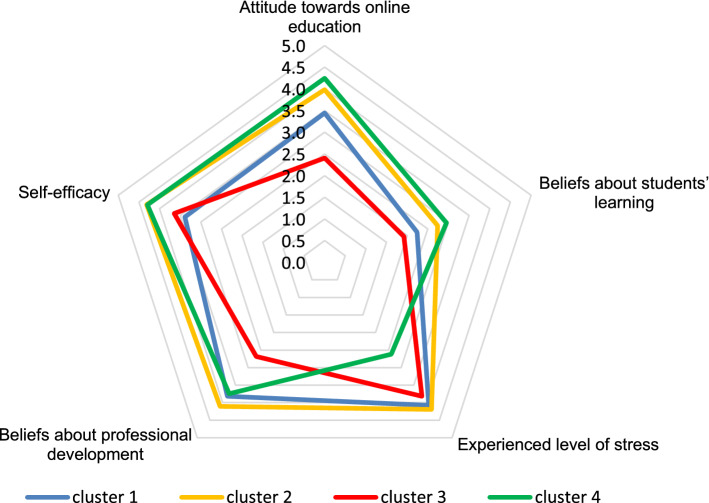
Radar graph of the clusters based on the means on the five cluster variables

For each cluster, the mean and standard deviation of the five variables were calculated (see Table 4) and a radar graph was plotted to highlight the differences between the means (see Fig. 1). Cluster 1 was the largest group (*n* = 110, 39% occurrence) and it was characterised by a relatively-high experienced level of stress and low self-efficacy. Cluster 2 (*n* = 60, 21% occurrence) was characterised by high values on all variables. Cluster 3 (*n* = 54, 19% occurrence) and was characterised by low values on all variables, except experienced level of stress and self-efficacy (both were moderate). Cluster 4 (*n* = 59, 21% occurrence) was characterised by high values on all variables except the experienced level of stress (low) and beliefs about professional development (moderate) (Table 5).

**Table 5:** ANOVA results for level of association between the clusters and the variables about the use and perception of support

Variable	df1	df2	*F*	*p*	Levene statistic	*p*	Welch statistic	*p*	Brown-Forsythe statistic	*p*
Perceived level of support	3	278	9.11	< .01*	7.68	< .001*	5.56	< 0.01*	8.10	< 0.01*
Satisfaction support services	3	250	7.54	< .01*	6.33	<0 .01*	6.46	< 0 .01*	6.91	<0 .01*
Use of support services	3	279	1.03	.38	1.04	0.38	0.92	0.43	1.04	0.38
Satisfaction trainings	3	181	0.90	.44	7.40	< 0.01*	0.70	0.55	0.73	0.54
Use of trainings	3	279	2.39	.07	1.41	0.24	2.75	0.05	2.43	0.07
Satisfaction tools	3	267	5.80	< .01*	6.51	< 0 .01*	4.17	0.01	5.18	< 0 .01*
Use of tools	3	279	2.64	.05*	1.15	0.33	2.86	0.04	2.59	0.05

More generally, two teacher profiles were relatively positive about online education (clusters 2 and 4) and two profiles were relatively negative about online education (clusters 1 and 3), based on teachers’ attitude towards online teaching and beliefs about students’ learning in online education. The positive group could be differentiated into a profile that experienced high levels of stress (Profile 2, ‘positive but stressed’) and a profile that experienced low levels of stress (Profile 4, ‘optimistic and easygoing’). The critical group consisted of a profile that experienced stress and had relatively low self-efficacy but believed that they learned a lot from the experience (Profile 1, ‘critical but eager’), and a profile that experienced somewhat less stress, had slightly higher self-efficacy and believed that they did not learn that much (Profile 3, ‘critical and reluctant’).

### Profiles and the use and perception of support

Table 6 shows the level of association among the clusters and the perceived level of support, the total use of support, the total use of training, the total use of tools, the average satisfaction about support, the average satisfaction about training and the average satisfaction about tools. A statistically-significant difference was found between clusters for the perceived level of support (*F*(3278) = 9.11, *p* < 0.01, η^2^ = 0.09), the average satisfaction about Edu-support (*F*(3250) = 7.54, *p* < 0.01, η^2^ = 0.08), the total use of tools (*F*(3264) = 2.39, *p* = 0.05, η^2^ = 0.03) and the average satisfaction with tools (*F*(3267) = 5.80, *p* < 0.01, η^2^ = 0.06). Moreover, there was a statistically nonsignificant but strong association between the clusters and the use of trainings (*F*(3279) = 2.39, *p* = 0.07, η^2^ = 0.03).

**Table 6:**
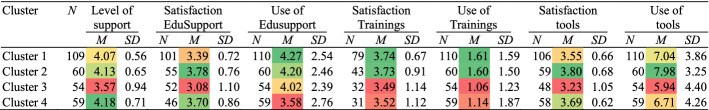
Number of teachers and mean and standard deviation for variables about use of and perception of support for each cluster

Post hoc Tukey tests (see Appendix 4) showed how the clusters differed across the variables (mean, standard deviation and number of teachers) by comparing pairwise differences between the clusters and the descriptive statistics (see Table 6). For all four variables that showed statistically-significant differences, we found differences between clusters 3 and 2 (i.e. Cluster 3 had lower values in terms of less use of services and lower satisfaction rates). Moreover, for all variables, except for the total use of tools, we found differences between clusters 3 and 4 (i.e. Cluster 3 had lower values). Besides these generic patterns, there was a statistically-significant difference between clusters 1 and 2 on satisfaction about Edu-support (Cluster 1 was lower), and between clusters 1 and 3 for the perceived level of support (Cluster 1 was higher).

Overall, teachers in Cluster 1 (critical but eager) made use of many education support services, teacher training opportunities and online tools, but they evaluated the education support services and teaching tools critically. Teachers in Cluster 2 (positive but stressed) made use of many education support services, teacher training opportunities and online tools, and evaluated these positively. Teachers in Cluster 3 (critical and reluctant) made little use of education support services, teacher training opportunities and online tools, and evaluated these critically. Teachers in Cluster 4 (optimistic and easygoing) made little use of education support services, online tools, and training, but evaluated these positively.

### Profiles and background characteristics

Table 7 shows a significant relationship between gender and the clusters, *χ*^2^ (6, *N* = 175) = 20,462, *p* = 0.002. To determine the pairwise relations, the z-scores were calculated based on the adjusted residual for each pair. Female teachers were overrepresented in Cluster 1 (critical but eager) and underrepresented in Cluster 3 (critical and reluctant). Males were overrepresented in Cluster 4 (optimistic and easygoing) and underrepresented in Cluster 1 (critical but eager). Teachers who preferred not to indicate their gender were overrepresented in Cluster 3 (critical and reluctant) and underrepresented in Cluster 4 (optimistic and easygoing).

**Table 7:** Chi-square test of independence for gender

Gender	Frequency	Cluster 1	Cluster 2	Cluster 3	Cluster 4	Total
Prefer not to answer	Count	7	3	10	0	20
	Expected count	8.0	4.1	3.8	4.1	20.0
	% within gender	35.0%	15.0%	50.0%	0.0%	100.0%
	Adjusted residual	− 0.5	− 0.7	3.8	− 2.4	
Male	Count	28	19	15	21	83
	Expected count	33.2	17.1	15.7	17.1	83.0
	% within gender	33.7%	22.9%	18.1%	25.3%	100.0%
	Adjusted residual	− 1.6	0.7	− 0.3	1.5	
Female	Count	35	14	8	15	72
	Expected count	28.8	14.8	13.6	14.8	72.0
	% within gender	48.6%	19.4%	11.1%	20.8%	100.0%
	Adjusted residual	1.9	− 0.3	− 2.2	0.1	
Total	Count	70	36	33	36	175
	Expected count	70.0	36.0	33.0	36.0	175.0
	% within gender	40.0%	20.6%	18.9%	20.6%	100.0%

Table 8 shows a significant relationship between teaching role and the clusters, *χ*^2^ (3, *N* = 268) = 8,943, *p* = 0.030. To determine the pairwise relations, the z-scores were calculated based on the adjusted residual for each pair. Lecturers were overrepresented in Cluster 3 (critical and reluctant) and Cluster 4 (optimistic and easygoing), whereas coordinators were overrepresented in Cluster 1 (critical but eager) and Cluster 2 (positive but stressed).

**Table 8:** Chi-square test of independence for teaching role

Teaching role	Frequency	Cluster 1	Cluster 2	Cluster 3	Cluster 4	Total
Lecturer	Count	55	30	35	40	160
	Expected count	62.1	34.6	29.3	34.0	160.0
	% within role	34.4%	18.8%	21.9%	25.0%	100.0%
	Adjusted residual	− 1.8	− 1.4	1.9	1.8	
Coordinator	Count	49	28	14	17	108
	Expected count	41.9	23.4	19.7	23.0	108.0
	% within role	45.4%	25.9%	13.0%	15.7%	100.0%
	Adjusted residual	1.8	1.4	− 1.9	-1.8	
Total	Count	104	58	49	57	268
	Expected count	104.0	58.0	49.0	57.0	268.0
	% within role	38.8%	21.6%	18.3%	21.3%	100.0%

The ANOVA on age and clusters showed no statistically-significant differences (*F*(3,111) = 0.319, *p* = 0.812).

A description of each profile is provided in Appendix 5 based on all the (distinctive) features.

## Discussion

### Interpretation of the profiles

The distinction between positive teachers (Profile 2 and 4) and critical teachers (Profile 1 and 3) corresponds with the education innovation literature (Kopcha et al., 2016; Scherer et al., 2020; Vocht & Laherto, 2021), and it could reflect more-general attitudes toward educational technologies (Garone et al., 2019; Jimoyiannis & Komis, 2007; MacIntyre et al., 2020). However, in this case, teachers’ attitudes toward online education are likely to be coloured by their experience of the crisis, such as the involuntariness of the transition to online education (Anderson et al., 2006; Debuse et al., 2008; Garone et al., 2019) and the experienced level of stress (Akour et al., 2020; Hew et al., 2020; Hodges et al., 2020). The collinearity tests that were performed prior to the cluster analysis (presented in Appendix 1) indeed showed a negative, or weak, correlation between stress and attitude towards online education and self-efficacy, which is in accordance with previous research (Herman et al., 2018; MacIntyre et al., 2020). However, the relations between the variables differed significantly for each teacher profile. For example, the teacher profile associated with the highest levels of stress was positive about online education and teacher self-efficacy (Profile 2, positive but stressed). Stress thus formed an important additional dimension in the clustering of teachers that is often absent in other innovation profiles (Garone et al., 2019; Kopcha et al., 2016; Scherer et al., 2020; Tao & Rosa Yeh, 2008a, 2008b). Because stress can provoke different cognitive and behavioural responses in teachers (MacIntyre et al., 2020), it should be interpreted in relation to the other psychological variables (self-efficacy and beliefs about professional development), as well as in relation to the perceptions about support and behavioural variables (the use of support services, teacher training and online teaching tools), which is discussed hereafter.

The profiles differed significantly in terms of teachers’ behaviour with regard to their teaching and professional development, as well as their perceived levels of and satisfaction about support. In general, attitudes toward online education are in accordance with the perception of support. However, the use of services (tools, training, support), which forms an indication of innovative teaching practices and professional development activities, corresponds more strongly with beliefs about professional development, especially if pairwise differences are taken into account. Profile 1 (critical but eager) was critical but made use of many services and differed most strongly from Profile 3 (the other critical profile) on beliefs about their professional development. Likewise, Profile 2 (positive but stressed) was less positive than Profile 4 (optimistic and easygoing) on all dimensions—except for beliefs about professional development—and made more use of services. A plausible explanation here is that the use of many services led to more-positive beliefs about professional development (i.e. teachers believed that they learned from using new tools, training and support services) (Thurlings et al., 2015). However, the relations with the other variables within each profile, such as the role of self-efficacy and stress, differed and could be explained by different factors that we did not investigate in this research. For example, the low self-efficacy of Profile 1 teachers (critical but eager) might reflect a self-critical attitude that could have increased their motivation for professional development and stimulated their use of support services—this hypothesis is based on the positive relation between importance, goal commitment and motivation (Ekinci & Acar, 2019; Lunenburg, 2011). In comparison, the high self-efficacy and positive attitude of Profile 2 teacher mighty have supported them to use services—this hypothesis is based on the positive relation between self-efficacy and innovative behaviour (see, for example, Runhaar & Sanders, 2016; Stylianidou et al., 2005). Moreover, stress can either activate or disactivate teachers to develop their competencies and innovate their teaching practices, depending on the type of stress (e.g. distress or eustress) and how they cope with or respond to these perceptions (e.g. avoidance or approach activities) (Akour et al., 2020; Federkeil et al., 2020; MacIntyre et al., 2020). This study showed that the teachers who experienced more stress (Profile 1 and 2) used more services and were more positive about their professional development in comparison with the profiles with a similar attitude towards online education (Profile 3 and 4, respectively). This contrasts with other studies that revealed a negative relation between stress and teachers’ behaviour change with regard to innovative teaching practices and professional development (Herman et al., 2018).

The teacher profiles significantly differed with regard to gender and teaching role (coordinator or lecturer). Female teachers were overrepresented in the ‘critical but eager’ profile. Results from earlier studies about the role of gender in teaching during COVID-19 are mixed (Akour et al., 2020; Federkeil et al., 2020; Hayes et al., 2020). In some studies, female teachers experienced more stress, but causal factors were attributed to different gendered roles, including the gendered role at home when working from home during COVID-19 (Federkeil et al., 2020; Hayes et al., 2020). Moreover, the activities and responsibilities linked to the coordinator role—such as communicating course activities with lecturers and students, a longer period of teaching, and feeling responsible for the course as a whole during the crisis–meant that these teachers were less likely to be ‘reluctant’ (Profile 3) or ‘easygoing’ (Profile 4). These results indicate that the profiles are partly shaped by the role of a teacher in this context, and thus do not reflect idiosyncratic teacher personalities (Göncz, 2017; Klassen & Tze, 2014; Perera et al., 2018). The profiles had no significant association with age, which is in accordance with the profiles of teachers’ readiness for online education in higher education (Scherer et al., 2020) and the mixed findings in the literature review about teachers’ innovative behaviours (Thurlings et al., 2015).

### Contributions, limitations and suggestions for future research

Overall, our results show that independent variables from a large teacher sample can consistently (significantly) and coherently (meaningfully) co-occur at certain levels within subgroups of teachers. Moreover, the relations between variables differed for each profile, which created idiosyncratic multidimensional profiles. Hence, the insights into the combinations of characteristics within individuals would not have transpired in a variable-oriented approach. This is because a positive correlation between two variables within one group and a negative correlation between two variables within another would cancel each other out. The multidimensionality of the profiles confirms that it is essential to account for the heterogeneity of teacher populations. This means research should carefully consider sampling procedures for teachers or take person-centred research approaches (e.g. cluster analyses, latent profile analyses, latent class analysis). This is particularly important when situations are new or unique, such as in crisis situations.

This study provides a starting point for such research, but it comes with several limitations. First, the profiles are based on a teacher sample of a single university, which hampers the generalisability of the profiles. Future research should include a larger and more-varied sample, as well as factors that help in understanding the role of the context. These could include the scientific discipline, cultural orientation, type of organisation or educational institution, broader educational or academic system and other country-related factors (Scherer et al., 2020). Second, this inductive, practice-oriented study used a limited number of questionnaire items to establish factors with acceptable reliability. Future research could use validated measures to establish more reliable factors, such as teachers’ psychological status (Akour et al., 2020). To better understand the role of the crisis (the Covid-19 pandemic) and the specific innovation (online education), it is important to further specify and differentiate the role of involuntariness in relation to motivation components (involuntariness can also play a role in other educational innovations that involve technology, such as the transition to a new LMS), stress and other psychological outcomes and coping activities. Moreover, in order to better interpret the differences between the ‘reluctant’ and ‘easygoing’ profiles, on the one hand, and the ‘eager’ and ‘stressed’ profiles, on the other, it is important to study how experienced stress and perceived efficacy relate to actual teaching abilities, practices or performances, including the effects on students. Third, besides including more and more-reliable factors in similar types of quantitative studies, qualitative inquiries (e.g. interviews) and interpretive analysis (e.g. phenomenological analysis) could help to increase our understanding about how teachers cope with challenges in a continuously changing context (i.e. how they interpret the circumstances and intend to improve the situation with their actions as part of an evolving meaning-making process).

### Implications for practice

Educational practice should consider differences between teachers and develop more-targeted forms of communication, support and policy to account for those differences. This study shows how some variables that co-occur within individuals and together form key dimensions on which teachers differ. Variables relevant in any educational change include teachers’ overall attitude toward educational change and education support, teachers’ experienced level of stress or psychological well-being more generally, teacher self-efficacy, and teacher use of various support services (training, teaching tools, support). Moreover, although the immediate COVID-19 crisis situation was unique, teachers probably will have to adapt their teaching more frequently and online tools are likely to play an important role. The profiles presented in this study can help to prepare for such a future.

There are two profiles that make little use of tools, training and support services (the ‘reluctant’ and ‘easygoing’ teacher profiles). These teachers have little contact with education support and are therefore easily overlooked. Moreover, they are relatively confident about their own efficacy in comparison with the profiles that are equally positive or critical, and thus they might not feel the need to work on their professional development or innovate their teaching practices. Some teachers could indeed be highly competent and need little support, but some others might have limited competencies and/or be unaware of the potential for improvement. The challenge is to identify and reach those teachers, as well as to motivate them to develop their teaching practice. Because self-efficacy is one of the stronger determining factors in teachers’ innovative behaviour (Thurlings et al., 2015), it is important to support the sense of efficacy while also pointing out any room for improvement. The ‘critical and reluctant’ profile (Profile 3) teachers might be particularly hard to reach because they have a negative perception about support. Teachers in Profile 1 are demanding in that they are critical towards themselves and support but are eager to learn. They need good educational and technical support. All profiles except for ‘easygoing’ teachers experience stress, which can have a negative impact on self-efficacy, teaching performance, burnout and depression (Lens & Jesus, 2010). Hence, a key challenge is to stimulate teachers to develop and innovate, while not increasing external pressure and stress levels. Externally forced educational changes, such as the move to online education during the Covid-19 crisis, can diminish feelings of voluntariness and autonomy. Providing the conditions for teachers to experience autonomy (giving choices), competence (positive performance feedback, optimal teacher support services) and relatedness (collaborative learning and innovation, solidarity) can help to increase intrinsic motivation and mitigate stress (Anderson & Iwanicki, 1984; Jansen in de Wal et al., 2014; Lens & Jesus, 2010).

## Conclusion

In this study, we identified four teacher profiles based on teachers’ attitudes towards online teaching, beliefs about students’ learning in online education, experienced level of stress, self-efficacy in online teaching, and beliefs about their professional development. Two profiles were relatively positive about online education and two profiles were relatively critical about it. The positive group consisted of a subgroup that experienced high levels of stress (‘positive but stressed profile) and a subgroup that experienced low levels of stress (‘optimistic and easygoing’ profile). The critical group of teachers consisted of a subgroup who experienced stress, had relatively low self-efficacy but believed that they learned a lot from the experience (‘critical but eager’ profile), and a subgroup who experienced somewhat less stress, had a slightly higher self-efficacy and believed that they did not learn that much (‘critical and reluctant’ profile).

Stress thus formed an important dimension in the clustering of teachers that is often absent in other teacher innovation profiles. Moreover, stress, self-efficacy and beliefs about professional development were related differently within each profile, resulting in multidimensional profiles. The teacher profiles that were identified based on psychological variables also differed significantly and meaningfully in their behaviour (use of tools, training and support), as well as their perceptions about support. Taken together, the results show that independent variables from a large teacher sample can consistently (significantly) and coherently (meaningfully) co-occur at certain levels within subgroups of teachers.

To account for the heterogeneity of teacher populations, education research should carefully consider sampling procedures for teachers or take person-centred research approaches (e.g. cluster analyses, latent profile analyses, latent class analysis). Educational practice should consider differences between teachers and develop more targeted forms of communication, support and policy.

## Appendix 1

See Table 9.

**Table 9:** Collinearity tests

Scale	Correlations				
	Beliefs about students’ learning	Experienced level of stress	Beliefs about professional development	Self-efficacy	Attitude towards online education
Beliefs about students’ learning	1.000	− 274	0.275	0.207	0.583
Experienced level of stress	− 274	1.000	0.023	− 123	− 224
Beliefs about professional development	0.275	0.023	1.000	0.099	0.398
Self-efficacy	0.207	− 123	0.099	1.000	0.301
Attitude towards online education	0.583	− 224	0.398	0.301	1.000

collinearity among variables, Spearman’s rho correlation

## Appendix 2

See Table 10.

**Table 10:** Multicollinearity VIF tests

Dependent variable: Beliefs about students’ learning		
Experienced level of Stress	0.921	1.085
Beliefs about professional Development	0.818	1.223
Self-efficacy	0.918	1.089
Attitude towards online education	0.722	1.384
Dependent variable: Experienced level of stress		
Beliefs about professional development	0.823	1.215
Self-efficacy	0.922	1.085
Attitude towards online education	0.552	1.812
Beliefs about students’ learning	0.630	1.587
Dependent variable: Beliefs about professional development		
Self-efficacy	0.919	1.088
Attitude towards online education	0.602	1.660
Beliefs about students’ learning	0.608	1.646
Experienced level of stress	0.894	1.118
Dependent variable: Self-efficacy		
Attitude towards online education	0.572	1.749
Beliefs about students’ learning	0.599	1.668
Experienced level of stress	0.879	1.137
Beliefs about professional development	0.807	1.239
Dependent variable: Attitude towards online education		
Beliefs about students’ learning	0.793	1.261
Experienced level of Stress	0.886	1.129
Beliefs about professional Development	0.890	1.124
Self-efficacy	0.961	1.040

VIF measures range between 1 and 2, all below the threshold of 2.6, confirming the lack of multicollinearity among the clustering variables (Galloway & Bretz, 2015; Sarstedt & Mooi, 2019).

## Appendix 3

See Table 11.

**Table 11:** Results and interpretation of cluster solutions

N clusters in solution	min. N cases per cluster	max. N cases per cluster	SD cases per cluster	Sum* F*	SD* F*	VRC	Highest Eta squared	Lowest Eta squared	SD Eta squared	*M* Eta squared
2	59	224	83	357.63	61		0.21	0.00	0.13	0.18
3	54	170	65	398.16	43	91	0.52	0.10	0.14	0.34
4	54	110	29	347.42	29	18	0.57	0.30	0.09	0.41
5	40	70	11	314.55	26	20	0.58	0.30	0.11	0.45

An agglomerative cluster analysis was applied to a cluster solutions range from two to five clusters, and an ANOVA was applied to generate statistics for each cluster solution. To compare the cluster solutions and decide about the most appropriate solution, we interpreted the number of cases in each cluster (no outliers and minimum variance in cluster size is favourable), the *F* value for each variable (low within group variance and high between group variance), the variance ratio criterium (VRC) to compare the sum total *F* of a cluster solution with the preceding and subsequent solution (maximum value is favourable), and the Eta squared for each variable, which indicates the proportion of the total variation in the cluster that can be attributed to the variable (high values for each variable are favourable).

The cluster solution of two clusters resulted in a small cluster (*N* = 59) that scores high on all variables except stress, and a large cluster (*N* = 224) that scores low on all variables except stress. This indicates that stress is negatively correlated with the other variables, as already indicated in the pre-test, Appendix 1. This reflects an overall positive or optimistic group and an overall negative or critical group. This first distinction into an overall positive and negative group was predictable (common in cluster analysis), and it did not provide much insight into the heterogeneity of teachers. The subsequent cluster solution of three clusters subdivided the large critical group into a more moderate critical group with high stress (*N* = 170) and a more extreme critical group with less stress (*N* = 54). This indicates that the negative relation between stress and other variables does not apply equally to all teachers (i.e. correlation is not linear). However, in this cluster, solution self-efficacy had a very low effect (η^2^ = 0.10). Moreover, the clusters were still uneven in seize (SD = 65). The cluster solution of four clusters was much more evenly distributed regarding cluster size (SD = 29), as well as in regard to the effect size of the different variables (all η^2^ are > 0.30). The cluster solution of four clusters still showed a positive and negative group in terms of attitude toward online teaching and beliefs about students’ learning, but the groups differed on experienced level of stress, beliefs about professional development and self-efficacy. The clustering step from four to five clusters resulted in a subdivision of one cluster into sub-clusters that differed similarly on all five variables (a moderate and an extreme group). Hence, each subsequent cluster solution differentiated groups on an additional variable up until four clusters. The cluster solution of four clusters was thus considered most appropriate and meaningful for distinguishing teacher profiles.

To test the stability and validity of this agglomerative cluster solution, a K-means cluster analysis with a pre-set of four clusters was applied. This resulted in similar clusters with only 6.3% difference in values, much less than the 20% threshold (Sarstedt & Mooi, 2019).

## Appendix 4

See Table 12.

**Table 12:** Post hoc Tukey tests for pairwise differences between the clusters

Variable	Clusters		Mean difference	SE	*p*
Level of support	1	2	− 0.06	0.11	0.95
	1	3	0.496	0.12	0.00
	1	4	− 0.106	0.11	0.78
	2	3	0.557	0.13	0.00
	2	4	− 0.046	0.13	0.98
	3	4	− 0.603	0.13	0.00
Satisfaction EduSupport	1	2	− 0.385	0.14	0.03
	1	3	0.307	0.14	0.14
	1	4	− 0.313	0.15	0.16
	2	3	0.692	0.16	0.00
	2	4	0.073	0.17	0.97
	3	4	− 0.620	0.17	0.00
Satisfaction tools	1	2	− 0.247	0.12	0.17
	1	3	0.320	0.13	0.06
	1	4	− 0.143	0.12	0.64
	2	3	0.566	0.14	0.00
	2	4	0.104	0.14	0.87
	3	4	− 0.462	0.14	0.01
Use of tools	1	2	− 0.947	0.63	0.44
	1	3	1.092	0.65	0.34
	1	4	0.324	0.64	0.96
	2	3	2.039	0.74	0.03
	2	4	1.271	0.72	0.29
	3	4	− 0.767	0.74	0.73

## Appendix 5 Profile descriptions

In the following profile descriptions, we highlight the features that are distinctive in relation to the other clusters. The labels are based on the co-occurrence of the most distinctive features.

## Profile 1: Critical but eager to learn

Teachers in this group have a relatively negative attitude towards online teaching and relatively negative beliefs about students’ online learning: they believe students’ learning is worse in online education. Moreover, the perceived level of support is low, and they evaluate the education support services and teaching tools critically. They experienced more stress than the average teacher and have a low self-efficacy. However, they followed trainings and used many tools, and they believe they have learned a lot. It thus seems that the stress and low self-efficacy causes these teachers to do better. Relatively, there are a lot of course coordinators and women in this group. We call these teachers “critical but eager to learn” because they are critical about online education, support and themselves, but engage in activities to learn (i.e. they make use of a lot of trainings and tools) and evaluate their own learning development positively.

## Positive but stressed teachers

Teachers in this cluster have a relatively positive attitude toward online teaching and relatively positive beliefs about students learning online (they believe students’ learning is not much worse in online education). However, they experienced high levels of stress (the highest levels of all clusters). They make use of many education support services, online teaching tools and trainings and they evaluate these positively. The level of support is also perceived positively. They have high self-efficacy and believe they have learned a lot from the experience. Relatively, there are a lot of course coordinators in this group. We call these teachers “positive but stressed” because they are positive about online education, support services and themselves, but experience the highest level of stress.

## Critical and reluctant teachers

Reluctant teachers have a negative attitude towards online teaching and negative beliefs about students’ online learning (they believe students’ learning is worse in online education). They make little use of education support services, online teaching tools and trainings and they evaluate these critically. The perceived level of support is also very low in this group. Overall, they are the most critical of online education and support services of all groups. However, they are not the most critical about their own efficacy. Moreover, they do not experience much stress compared to the other groups. Most significantly, these teachers believe they do not learn much from the experience. Hence, in comparison to the other critical group (Cluster 1), they experience less stress, are more confident about their own efficacy, believe they learn less and make significantly less use of support, trainings and tools. When we look at the associations with background characteristics, we find that, relatively, this group of teachers has a lot of course coordinators and that many teachers preferred not to indicate their gender. We call these teachers “reluctant” because they are the most critical about online education and support, do not experience much stress, make little use of services, trainings and tools and they do not believe they learned from the experience.

## Optimistic and easy-going teachers

Teachers in this cluster have a positive attitude towards online teaching and positive beliefs about students’ learning online. They experienced low levels of stress (the least stress of all clusters). They did not make much use of education support services, online teaching tools and trainings. The perceived level of support was high. Relatively, there are a lot of male teachers in this group and a relatively high number of lecturers (rather than course coordinators). We describe these teachers as “optimistic and easy-going” because of their overall positive attitude, low levels of stress and low use of services (support, tools, trainings).

## Data Availability

The dataset and output from all empirical analyses are securely maintained. Anonymised data and output materials can be requested from the authors.
